# Analyzing Mandibular Characteristics for Age and Gender Variation Through Digital Radiographic Techniques: A Retrospective Study

**DOI:** 10.7759/cureus.58500

**Published:** 2024-04-17

**Authors:** Abirami Arthanari, Shanmathy Sureshbabu, Karthikeyan Ramalingam, Vignesh Ravindran, Lavanya Prathap, Prashanthi Sitaraman

**Affiliations:** 1 Department of Forensic Odontology, Saveetha Dental College and Hospitals, Saveetha Institute of Medical and Technical Sciences, Saveetha University, Chennai, IND; 2 Department of Oral Pathology and Microbiology, Saveetha Dental College and Hospitals, Saveetha Institute of Medical and Technical Sciences, Saveetha University, Chennai, IND; 3 Department of Pedodontics and Preventive Dentistry, Saveetha Dental College and Hospitals, Saveetha Institute of Medical and Technical Sciences, Saveetha University, Chennai, IND; 4 Department of Anatomy, Saveetha Medical College and Hospitals, Saveetha Institute of Medical and Technical Sciences, Saveetha University, Chennai, IND; 5 Department of Oral Medicine and Radiology, Saveetha Dental College and Hospitals, Saveetha Institute of Medical and Technical Sciences, Saveetha University, Chennai, IND

**Keywords:** projective ramus height, maximum ramus breadth, antegonial depth, bigonial width, mandibular metrics, gender determination, age estimation

## Abstract

Background

Forensic odontology has emerged as a crucial method for identifying skeletal or dental remains. Due to the restricted accuracy of current techniques for estimating age at death, researchers have endeavored to illustrate age-related alterations in dental hard tissues. Bone remodeling constitutes an ongoing and intricate process throughout our lifespan. It is believed that morphological changes in the mandible during an individual's lifetime are influenced by both dental condition and the individual's age.

Objectives

This study aims to evaluate the efficacy of mandibular parameters such as projective ramus height (PRH), maximum ramus breadth (MaRB), bigonial width (BGW), antegonial depth (Ant.D), and antegonial angle (Ant.A), as a gender-determining tool and compare and analyze the mandibular parameter measurements using digital orthopantomography.

Methodology

In this study, the total number of samples used was 500 out of which 250 were males and 250 were females. Planmeca software (Version 6.0, Planmeca Romexis, Charlotte, NC) was used and the accuracy test, analysis of variance (ANOVA), multiple regression, and discriminant analysis for gender were performed using SPSS for Windows, Version 16.0 (SPSS Inc., Chicago, IL).

Results

For age estimation, the least standard error of 0.008 was observed for BGW. A maximum standard error of 0.230 was observed for Ant.D. For sex determination, the coefficient function was positive for PRH, BGW, and Ant.A, with values of 0.202, 0.805, and 0.052, respectively. Ant.D and MaRB both exhibited negative values of -0.204 and -0.379, respectively.

Conclusions

Out of all the parameters assessed, BGW is the most preferred parameter for age estimation and Ant.A can be preferred for sex estimation. Age and gender can be estimated using the two parameters in the Indian population.

## Introduction

An important field of study in forensic anthropology is the determination of age and sex by anthropometric measurements of mandibular characteristics. This method provides a precise and accurate way to determine an individual's biological profile from their skeletal remains [1]. One of the main skeletal components used to estimate an individual's age is the mandible, which experiences unique morphological changes throughout a person's life. Its natural sexual dimorphism, which is the foundation for sex determination, adds to its significance as a biometric tool [2]. This study investigates the significance, techniques, and improvements in the use of mandibular traits for anthropometric age and sex evaluation. Anthropometry is very useful in forensic settings where skeletal remains are frequently valuable and delicate because it is a nondestructive procedure that maintains bone integrity.

The evolution of the human mandible is predictable, with distinct morphological changes occurring at various periods of life. The wear and eruption of teeth are two of the main markers of age [3]. Examination of a tooth's presence, absence, or condition might reveal important information about a person's age. As people age, their dental features and mandible structure change [4]. The mandibular symphysis, sometimes known as the midline junction, is a crucial site for age determination. A gradual union of the symphysis occurs during childhood and adolescence. Certain skeletal characteristics, like tubercles and mental spines, as well as the degree of fusion, can help with accurate age assessment [5].

Mandibular parameters are used to estimate an individual's gender by investigating the sexual dimorphic features of both males and females [6]. These features include the mandible's strength, form, and size. Compared to female mandibles, male mandibles are frequently larger and stronger. The ramus, or vertical region of the mandible, is typically wider in men than in women [7]. Mandibular angle is yet an important variable in determining sex. Males typically have more prominent square-shaped angles, whereas females tend to have more rounded and obtuse angles. Male mandibles tend to be larger, with evident attachments to muscle sites, and they are slightly more robust than female mandibles. Several nonmetric characteristics of the mandible have been linked to sex. Male characteristics include gonial flare, a broad ascending ramus, a prominent symphysis, and a modest mental eminence. These differences arise from the impact of hormones on adolescent development of bones [8].

Beyond these evident characteristics advancements in technology have enabled more precise measurement and analysis. Comprehensive assessments of mandibular structures are possible with the use of computed tomography (CT) scans and other three-dimensional imaging techniques [8,9]. One automated method that helps quantify minute changes in size and form and enhance the accuracy of determining age and sex is called geometric morphometrics. Considering that while mandibular metrics provide valuable information, a comprehensive approach often incorporates statistical models and the combining of numerous skeletal characteristics. Experts in anthropology and forensics consider the potential influence of population-specific variations, recognizing that specific populations may exhibit distinct variations in mandibular morphology [10].

In forensic contexts, estimating an individual's age and gender is essential since this aids in identifying their biological features. This information assists the work of law enforcement and forensic experts by supporting the investigation of crimes, finding missing individuals, and offering a sense of closure to families [3]. The dynamic area of investigating mandibular characteristics for age and sex determination blends the most advanced osteological techniques with current technology. A person's unique mandibular changes throughout their life provide forensic professionals with informative data that supports interdisciplinary efforts to solve complex crimes and give families and communities closure [11]. Recent studies have investigated whether radiographic images may indicate gender and age. This research employs mandibular measures, such as the projective ramus height (PRH), maximum ramus breadth (MaRB), bigonial width (BGW), antegonial depth (Ant.D), and antegonial angle (Ant.A), to assess sexual dimorphism in the South Indian population using panoramic radiographs. This study aims to evaluate the efficacy of mandibular parameters as a gender-determining tool and to compare and analyze the mandibular parameter measurements using digital orthopantomography.

## Materials and methods

Utilizing samples obtained from the archive of the Department of Oral Medicine and Radiology at Saveetha Dental College and Hospital, the current study was conducted within the Department of Forensic Odontology. A total of 500 samples, distributed equally between 250 males and 250 females aged between 41 and 50 years, were subjected to analysis. Orthopantomogram (OPG) radiographs with known sex, excellent clarity, and adequate contrast without any abnormalities in development were included in the study. The evaluation excluded radiographs that indicated pathological abnormalities or distortions. The project was approved by the Institutional Human Ethics Committee of Saveetha Dental College (IHEC/SDC/FACULTY/22/FO/059). The results were entered into an Excel for further statistical analysis once the samples were measured using Planmeca software (Version 6.0, Planmeca Romexis, Charlotte, NC). 

**Figure 1:**
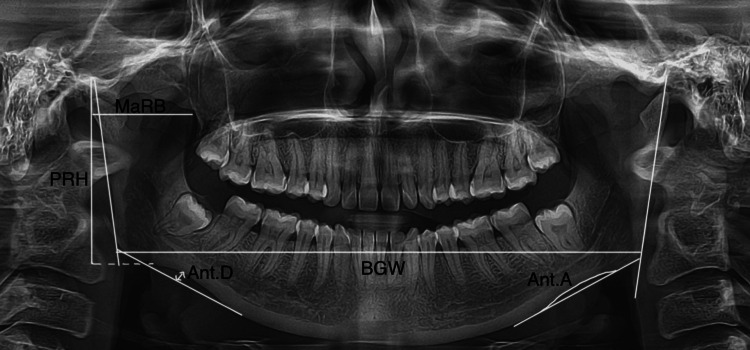
Represents parameters of projective ramus height (PRH), bigonial width (BGW), antegonial depth (Ant.D), antegonial angle (Ant.A), and maximum ramus breadth (MaRB).

All results were prepared using SPSS for Windows, Version 16.0 (SPSS Inc., Chicago, IL). In this study accuracy test, analysis of variance (ANOVA), multiple regression, and discriminant analysis for gender were performed on an individual. The parameter examined was the distance between the mandible's inferior surface and the mandibular condyle's highest point, known as the PRH. The BGW is a measure of the separation between two gonions. The term Ant.D refers to the distance measured on a perpendicular line from the inferior border of the mandible to the lowest point of the notch concavity. The measurement of the Ant.A is made using two lines that are parallel to the antegonial region and cross at the deepest point of the antegonial notch. The greater anterior-posterior diameter of the ramus serves as the basis for calculating the maximum ramus breadth (MaRB) as seen in Figure 1.

## Results

The analysis of mandibular parameters, including PRH, BGW, Ant.D, Ant.A, and MaRB, was conducted. The means of all the parameters on their combined means within the category (40-50 years) are illustrated in Table 1.

**Table 1:** The distribution of accuracy of prediction of sex based on various mandibular measurements (combined measurements). In this table, for the age group of 41-50 years, 89.4% of the original group were correctly classified as male and female.

Gender	Predicted group		Total
	Male	Female	
Male	230	20	250
Female	35	215	250
Gender	Prediction accuracy (%)		
Male	92.4%	7.6%	100%
Female	13.7%	86.3%	100%

The gender differences in mandibular shape were evaluated based on the division of the sample only by sex without including age as a factor. Considering the degree of freedom (df), analysis of variance (F), and the corresponding significance level (*P*-value) demonstrate the significance of the regression model, as shown in Table 2.

**Table 2:** Combined results of analysis of variance (ANOVA) applied to males and females. The table represents the *P*-value of 0.00, which is statistically significant for the residual model, and the *P-*value for regression is 0.349, which is statistically not significant.

Model	Sum of squares	Degree of freedom (df)	Mean square	Analysis of variance (F)	*P*-value
Regression	81.976	9	9.108	1.116	0.349
Residual	3982.522	488	8.161	0	0.00
Total	4064.498	497	0	0	0.00

In all these parameters, BGW had the least standard error of 0.008. Ant.D was found to have the highest standard error of 0.230. A negative *t*-value (ratio) was observed for PRH, MaRB, and Ant.D. In Table 3, BGW and Ant.A were observed to have positive *t*-values of 0.586 and 1.419, respectively, and their corresponding *P*-values were statistically not significant.

**Table 3:** Multiple regression analysis to calculate the age. The formula derived to analyze the PRH is age = 45.402 - 0.015*PRH. The formula derived to determine the MaRB is age = 45.402 - 0.026*MaRB. The formula derived to analyze the BGW is age = 45.402 + 0.005*BGW. The formula derived to analyze the Ant.D is age = 45.402 - 0.354*Ant.D. The formula derived to analyze the Ant.A is age = 45.402 + 0.102*Ant.A

Model	Unstandardized coefficient	Standardized coefficient	Std. error	Confidence (*t*-value)	*P*-value
						Constant (45.402)					
Projective ramus height (PRH) (mm)	-0.015	-0.028	0.025	-0.606	0.545
Maximum ramus breadth (MaRB) (mm)	-0.026	-0.036	0.034	-0.744	0.457
Bigonial width (BGW) (mm)	0.005	0.029	0.008	0.586	0.558
Antegonial depth (Ant.D) (mm)	-0.354	-0.232	0.230	-1.537	0.125
Antegonial angle (Ant.A) (°)	0.102	0.213	0.072	1.419	0.157

**Figure 2:**
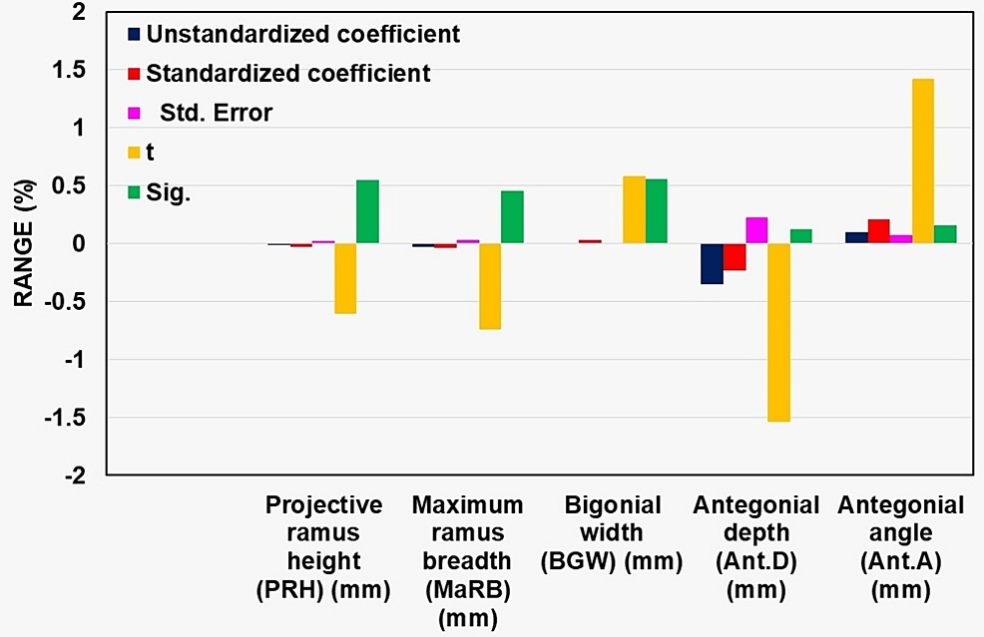
The bar chart represents the multiple regression analysis.

In Table 3, the unstandardized coefficients, the lowest value was observed in Ant.D (-0.354) and the highest value was observed in Ant.A (0.102). For standardized coefficients, the lowest value was observed in Ant.D (-0.232) and the highest value was observed in Ant.A (0.213). A standard error of 0.025 for PRH, 0.034 for MaRB, 0.008 for BGW, 0.230 for Ant.D, and 0.072 for Ant.A was observed. A positive *t*-value of 0.586 and 1.419 was observed for BGW and Ant.A, respectively, as illustrated in Figure 2

Out of all the parameters considered, PRH, BGW, and Ant.A showed a positive coefficient function of 0.202, 0.805, and 0.052, respectively. MaRB and Ant.D showed a negative value of -0.379 and -0.204, respectively. The regression formula derived for gender analysis is 0.202*PRH - 0.379*MaRB + 0.805*BW - 0.204*Ant.D + 0.052*Ant, as given in Table 4.

**Table 4:** Discriminant analysis for gender.

Mandibular parameters	Function 1
		Projective ramus height (mm)	0.202
Maximum ramus breadth (mm)	-0.379
Bigonial width (mm)	0.805
Antegonial depth (mm)	-0.204
Antegonial angle (°)	0.052

## Discussion

In our research, age estimation shows the very least standard error of 0.008 in BGW and a maximum standard error of 0.230 observed in Ant.D. Also, the *t*-value of 0.213 and 0.029 was observed for the Ant.A and BGW, respectively. In gender analysis, the coefficients of 0.202, 0.805, and 0.052 were shown positive for PRH, BGW, and Ant.A. Ant.D and MaRB both obtained negative values of -0.204 and -0.379, respectively. Out of all the parameters assessed, BGW can be best preferred for age estimation and Ant.A can be preferred for sex estimation.

The determination of age and gender based on mandibular traits is an important area in both forensic odontology and anthropology. The mandible, the largest and strongest bone in the skull, can provide vital information about a person's age and gender, especially in forensic instances where other methods of identification may be limited [12]. One of the most common methods for calculating age from mandibular information is to examine dental development and eruption patterns. Teeth erupt and develop in a predictable order during development and adolescence [13]. Using dental age standards as a guide, forensic experts can determine an individual's chronological age by examining their dental growth stages. This is especially useful when dealing with juvenile remnants or when age-associated evidence is necessary for the course of the investigation [14].

OPGs are commonly recommended and widely utilized by medical professionals as an appropriate screening technique for the identification of dental conditions [15]. It is considered an adjuvant radiographic technique to identify gender because it enables the measurement of multiple landmarks from skeletal remains. Panoramic images have several advantages, including their broad coverage, low patient radiation dosage, and quick capture. Other advantages include the lack of interference from overlaid images. In addition, image contrast and brightness improvement, as well as enlargement, give an accurate and reliable technique for measuring particular locations [16].

In forensic anthropology, comparing postmortem and antemortem radiographs is one of the most significant processes in verifying the identity of human skeletal remains [17]. Furthermore, when only one characteristic is available, such as partial or mutilated mandibles, evaluating the demarking point plays a significant role in determining gender. The means of the variables show that males had higher minimum and maximum ranges than females. Thus, statistically, one can ascertain if the offered sample is a male or a female by assessing it with the indicated dimension and referring to the distinguishing point [18].

When it involves skeleton sex determination, metric research studies on radiographs are frequently found to be superior in terms of objectiveness, accuracy, and reliability. In forensic anthropology, a calibrated measurement tool was employed, and antemortem radiographs are the foundation for identifying human remains [19]. The abundance of panoramic radiography provides an excellent chance to research sexual dimorphism and age estimation of individuals in specific populations. The sex of an unknown individual can be identified using information from the morphology and metric aspects of the skull and mandible, soft tissues, dental records, and DNA analysis of teeth [20].

Mandibular factors are positive, but it is critical to be aware of any potential downsides and differences in population-specific features that may alter the accuracy of the results. Because these variations could be caused by genetic, environmental, or dietary factors, comprehensive databases and standards covering a wide range of people are required to properly predict age and sex based on mandibular traits [20]. The mandibular ascending ramus height is the vertical distance between the mandibular notch and the highest point of the mandibular condyle. Differences in this dimension may indicate sexual dimorphism, allowing sex to be identified. MaRB, the biggest lateral dimension of the mandibular ramus, is an essential anatomical parameter that is essential for dental anthropology, forensic investigations, and anatomical studies [21].

Bigonial breadth affects facial harmony and symmetry, as it is measured at the largest parts of the mandible and is frequently investigated in anthropology for understanding population dynamics and evolutionary patterns [22]. Ant.D is the distance between the antegonial notch and the mandible's inferior border. Ant.A is calculated by drawing two lines parallel to the antegonial region and intersecting at the deepest point of the antegonial notch. Age-related processes, such as bone resorption, may change the Ant.A [23,24].

According to Leversha et al., BGW shows significant measures to use when estimating an individual's age, except for some age group comparisons where the difference was not statistically significant [13]. Except for the gonial angle, all the factors that Esfehani et al. included in their study can be used as a helpful tool for sex estimation [25]. Ant.A may be a positive index for sex assessment, according to a study done by Apaydin and Ozbey, on the prepubertal Turkish population [26]. In our study, it becomes clear that the findings are consistent with those of earlier investigations. A statistically significant gender difference was seen for gonial angle, ramus height, and BGW. Bhuyan et al., their study explained that the parameters increased with an increase in age. This difference was statistically significant on the right side for gonial angle and ramus height, which used panoramic radiographs to determine an individual's age and sex [2].

Examining these modifications makes it easier to establish an individual's age. Men often have bigger angles than women. This distinction is useful for determining sex during forensic investigations [24]. The use of mandibular features to determine age and sex has significant consequences for forensic anthropology and odontology. Forensic professionals can identify a person in forensic investigations using a variety of techniques, including mandibular morphological assessment and dental development analysis [26]. As technology advances, the field should expect further methodological developments as well as an improved ability to effectively extract information from skeletal remains, which will aid in the resolution of forensic cases throughout the world.

Limitations

The analysis of mandibular parameters, including PRH, BGW, Ant.D, Ant.A, and MaRB, exhibited notable variations among these measurements. These variations may pose limitations in accurately assessing or generalizing findings related to mandibular morphology or function. Therefore, careful consideration and potentially additional investigations are warranted to address and mitigate these variations in future analyses or interpretations.

## Conclusions

In summary, the use of OPG imaging to determine age and gender based on mandibular parameters has shown to be a useful and adaptable method in a variety of fields, such as clinical diagnostics, forensic anthropology, and dentistry. The mandible is a prominent facial bone that changes constantly throughout a person's life, making it a good indicator of gender and age. Forensic situations can benefit from the potential for age prediction using mandibular characteristics. OPG scans are used by forensic anthropologists to determine the age at death of unidentified skeletal remains. The accuracy and dependability of age and gender estimate techniques should be further improved by interdisciplinary research collaborations and ongoing improvements in imaging technology.
